# Equity and healthy ageing

**DOI:** 10.2471/BLT.16.187609

**Published:** 2017-09-18

**Authors:** Sridhar Venkatapuram, Hans-Jörg Ehni, Abha Saxena

**Affiliations:** a Department of Global Health & Social Medicine, King’s College London, Strand, London, WC2R 2LS, England.; b Institute for Ethics and the History of Medicine, University of Tübingen, Tübingen, Germany.; c Global Health Ethics Unit, World Health Organization, Geneva, Switzerland.

In many areas of the world, old age is perceived as an end-of-life stage characterized by declining physical and mental faculties, increased risk of morbidity and withdrawal from productive social activities. Interestingly, the chronological age at which a person is first considered to be old varies across societies^1^^,^^2^ and appears to be positively correlated to life expectancy in a particular global region or society. The dramatically different life expectancies across societies mean remarkably varied starting points for old age.^3^

Alongside research on the great global diversity in conceptions of old age, an important body of scientific research has been examining whether chronological age is, in fact, linked to physical and mental decline, as generally assumed. Surprisingly, only a weak correlation has been observed.^4^ Other aspects of an individual’s life – e.g. behaviours, genetic inheritance, and, most substantially, social factors throughout the life course – appear to be more strongly correlated with physical functioning and quality of life in old age.^5^ In terms of what older persons can do, some individuals aged 60 years or older are similar to 20- to 30-year-olds until quite near their deaths. The commonly-held view that old age is a period of declining faculties that is natural and inevitable is therefore not wholly accurate and has hidden preventable inequalities in the quality of life of older people. Importantly, the causal role of social conditions in the great variations in the quality of life of older individuals within a country – as well as across countries – raises profound questions of social justice and social action. Perhaps the most important questions to be answered are: (i) are the differences or inequalities observed in abilities and quality of life among older persons unfair and unjust; (ii) if the observed differences or inequalities are unfair and unjust, how are they unfair and unjust; and (iii) if there is unfairness and injustice, what are the right and required national and global responses.

Philosophical discussions over the past two decades about the ethics of health inequalities have led to recognition that the concern for health equity is multidimensional.^6^ We should expect the same regarding health inequalities in old age. For example, we would not want to level-down the health of some in order to achieve equal health outcomes. The ethical importance of health and the concern about health inequalities lead us to evaluate multiple dimensions – e.g. causes, consequences, differences in experiences and distribution patterns – in order to see where, when and what kind of social response is required. Importantly, these multiple dimensions have to be judged or evaluated against some ethical standard that can give guidance for social action. The traditional bioethics principles drawn from moral philosophy are not enough. While they can help guide individual actions, we need guidance for social and global action that may have to target many social determinants of health and health inequalities within and across countries.^7^ Social epidemiology extends the scope of analyses far beyond individual behaviours, biology and health care to the functioning of basic social institutions and practices at local to global levels.^8^^,^^9^ The complementary ethical evaluation of the functioning of institutions and practices needs to be based on political philosophy and theories of social and global justice – including human rights.^10^^–^^12^ Such theories must give a central place to health if they are going to be helpful.

So far, philosophical discussions on health equity have given little attention to the issues of healthy ageing and health inequalities among older people. Ethicists have previously considered the balance between the provision of health care for older people and the health resources offered to younger people – i.e. so-called intergenerational equity^13^ – as well as some of the ethical aspects of dementia care and life-extension technologies.^14^ However, little consideration has been given to the inequalities in health and well-being among older people within and across countries. There has also been little examination of the ethical implications of such differences when traced to the social conditions operating at various points in an individual’s life and or cumulatively over the individual’s life. In fact, the current views on health-care priority setting may be affected if it is recognized that, because of its multiple disadvantages, a particular socioeconomic group of older individuals merits a disproportionate amount of the available health care. If global and social conditions are creating inequalities in health and well-being that are very specific to individuals in their old age, then the ethical problem extends far beyond priority setting in health care. Doing justice to older people requires that we identify the right social response to prevent and correct the direct harm that broader social conditions have done to those people over their entire life course – as well as during old age. Such demands of justice do not seem reducible to a priority-setting exercise in resource allocation.

In 2015, the World Health Organization (WHO) published its *World report on ageing and health*.^5^ This report brought to global attention a whole range of issues related to the health and well-being of older individuals and populations across the entire spectrum of high- middle- and low-income countries. Aside from being an important resource for the planners and implementers of health policies, the report was ethically important in laying the groundwork for identifying and addressing potential injustices experienced by older people worldwide. In particular, it highlighted two neglected issues: elder abuse and the impact of emergency situations on older people. The report’s most important link between ageing and social justice is its novel definition of healthy ageing – i.e. having “functional abilities to be and do what an older person has reason to value”.^5^ The functional abilities include being able to have a role or identity, to have relationships and to have the possibility of autonomy, enjoyment, potential for personal growth and security. These abilities are not just personal attributes. Instead, they represent the combined interaction of a person’s so-called intrinsic capacity and external environmental conditions. Regardless of the decreases in intrinsic capacity that are common features of old age, environmental conditions can support older people and help keep functional abilities at a decent or high level. By making surrounding social conditions constitutive of functional abilities, the health of older people becomes a matter of social choice and action.

The report acknowledges that the functional-ability concept of health is very similar to the health-capability concept being developed by advocates of a theory of social and global justice called the capabilities approach.^10^ Whether by coincidence or foresight, the report places the ethical tools strategically right next to the problem. The WHO’s Global Health Ethics Unit is beginning to investigate how the capabilities approach might function as a general ethical framework to help create an age-friendly world, as well as provide specific guidance for particular issues, such as age-based rationing, dementia care and elder abuse.

 Social action toward improving the quality of life of older people as well as old age equity is also urgent and unavoidable because of population ageing – i.e. the rapidly growing number of older people worldwide and, particularly, in developing countries. Between 2000 and 2015 there were substantial gains in life expectancy in all the regions of the world.^15^ At a global level, a human being born between 2010 and 2015 can expect to live a mean of 70.8 years – or 3.6 years longer than an individual born between 2000 and 2005.^15^ Over the same period, the percentage of the population in each region made up of people aged 60 years or older also increased and – in all regions except Africa – is expected to reach 25% or more by 2050.^15^ It has been predicted that the number of older people – estimated to be 962 million in 2015 – will rise to 2.1 billion by 2050, with 80% of such people then living in developing countries.^15^ There are many major challenges to be faced by a whole array of actors. This includes individuals, families and communities to companies, national governments and international organizations, if we are to ensure that all of those additional years lived are healthy and of good quality. Although global population ageing may be well underway and beyond policy levers, the reduction of inequalities in the care and quality of life of older people is very much within social control and possible in all countries. Foremost on the agenda for action must be the identification and mitigation of the worst injustices being done to older people. Injustices that have gone unrecognized due to our incorrect and ill-informed assumptions about human ageing.
